# CaMKII binding to GluN2B is important for massed spatial learning in the Morris water maze

**DOI:** 10.12688/f1000research.4660.1

**Published:** 2014-08-12

**Authors:** Ivar S. Stein, Michaela S. Donaldson, Johannes W. Hell

**Affiliations:** 1 Department of Pharmacology, School of Medicine, University of California, Davis, 95616-8636, USA

## Abstract

Learning and memory as well as long-term potentiation (LTP) depend on Ca ^2+^ influx through the NMDA-type glutamate receptor (NMDAR) and the resulting activation of the Ca ^2+^ and calmodulin-dependent protein kinase (CaMKII). Ca ^2+^ influx via the NMDAR triggers CaMKII binding to the NMDAR for enhanced CaMKII accumulation at post-synaptic sites that experience heightened activity as occurring during LTP. Previously, we generated knock-in (KI) mice in which we replaced two residues in the NMDAR GluN2B subunit to impair CaMKII binding to GluN2B. Various forms of LTP at the Schaffer collateral synapses in CA1 are reduced by 50%. Nevertheless, working memory in the win-shift 8 arm maze and learning of the Morris water maze (MWM) task was normal in the KI mice although recall of the task was impaired in these mice during the period of early memory consolidation. We now show that massed training in the MWM task within a single day resulted in impaired learning. However, learning and recall of the Barnes maze task and contextual fear conditioning over one or multiple days were surprisingly unaffected. The differences observed in the MWM compared to the Barnes maze and contextual fear conditioning suggest a differential involvement of CaMKII and the specific interaction with GluN2B, probably depending on varying degrees of stress, cognitive demand or even potentially different plasticity mechanisms associated with the diverse tasks.

## Introduction

The acquisition and storage of new tasks as well as the modification of already existing memories depend on the selective strengthening and weakening of synaptic interactions embedded in extensive networks of neurons ( Kessels & Malinow, 2009; Martin *et al.*, 2000; Morris, 2013; Neves *et al.*, 2008). Synaptic plasticity and especially long-term potentiation (LTP), a stable increase in synaptic strength, are emerging as the cellular equivalent of learning and memory ( Gruart *et al.*, 2006; Whitlock *et al.*, 2006). Two key players involved in LTP are the Ca ^2+^/calmodulin-dependent protein kinase II (CaMKII) and the NMDA-type glutamate receptor (NMDAR). Ca ^2+^ influx through the NMDAR leads to CaMKII activation and recruitment to the postsynaptic density (PSD), which can be persistent and synapse specific ( Otmakhov *et al.*, 2004; Zhang *et al.*, 2008) (but see also ( Rose *et al.*, 2009)). CaMKII binding to aa 1290–1309 on the NMDAR GluN2B subunit is required for this activity-dependent translocation ( Halt *et al.*, 2012; Leonard *et al.*, 1999; Strack & Colbran, 1998; Strack *et al.*, 2000) and is crucial for LTP ( Barria & Malinow, 2005; Halt *et al.*, 2012; Zhou *et al.*, 2007). Stimulation of CaMKII results in its auto-phosphorylation on T286 causing a persistent Ca ^2+^-independent activation of CaMKII ( Lisman *et al.*, 2002). This auto-phosphorylation is increased after LTP and spatial learning in the Morris water maze (MWM) ( Lengyel *et al.*, 2004; Ouyang *et al.*, 1997; Ouyang *et al.*, 1999; Tan & Liang, 1996) and is required for effective binding of CaMKII to GluN2B ( Bayer *et al.*, 2001; Strack & Colbran, 1998). T286A CaMKII mutant mice exhibit no hippocampal NMDAR dependent LTP and have impaired MWM learning ( Giese *et al.*, 1998).

We recently reported the specific disruption of CaMKII binding to GluN2B by point mutations in the GluN2B gene ( Halt *et al.*, 2012). The GluN2BKI mice contain two point mutations in the GluN2B C terminus (L1298A and R1300Q). Each of these mutations abolishes CaMKII binding to this site nearly completely *in vitro* ( Strack *et al.*, 2000). *In vivo* the two point mutations entirely abrogate the activity-dependent increase in the interaction with the NMDAR, show reduced hippocampal LTP by 50% and result in a MWM memory deficit, while acquisition of the MWM task and working memory as evaluated in the 8 arm win shift test remain normal ( Halt *et al.*, 2012).

Now we report a specific moderate spatial learning deficit in the GluN2B knock in (KI) mice during massed 1-day training in the MWM. At the same time learning and memory recall in the Barnes maze and contextual fear conditioning are unaffected.

## Material and methods

### Mice

All animal procedures were approved (Protocol #: 15512; PHS/NIH Assurance A3433-01) by the UC Davis Institutional Animal Care and Use Committee (IACUC) and followed NIH guidelines. All experiments were conducted with litter matched wild type (WT) and GluN2B KI mice of mixed sex (evenly distributed gender ratios) and between 2 and 4 months of age; mice within a cohort were not more than 3 weeks apart. For a more detailed characterization and description of the GluN2B KI mice (C57BL/6J background) including genotyping procedures refer to ( Halt *et al.*, 2012). The mice were on a 12 h light/dark cycle, housed individually for the behavioral experiments and acclimated for at least one week before all procedures. After the acclimation period and before the training and testing the mice were extensively handled (7–10 times for 1–2 min on different days). All trainings and testing were performed during the 12 h light cycle.

### Morris water maze

The MWM was a circular, enamel coated steel tank, 94 cm in diameter filled with water at 22–24°C. For the visible trial, a black labeled square platform (6 × 6 cm) emerging 2 cm out of the opaque (through addition of non-toxic paint) water surface was used, while the clear Plexiglas square platform (6 × 6 cm) for the training trials was submerged 2 cm below the opaque water surface. On the first day, the mice were trained in one visible trial followed by 12 consecutive training trials, while the platform was kept in a fixed position. The mice were always placed facing the wall of the pool and started randomly from three different starting positions that are equally distributed around the perimeter and are not located within the target quadrant. The mice were allowed to swim freely for 90 s to find the platform. If they failed to locate the platform within time, they were gently guided to it. After the mice climbed on the platform they were allowed to remain 30 s on the platform before they were removed from the pool and placed in their home cage for an inter-trial interval of 6–10 min. The mice were returned to the pool for a 90 s probe test without the platform 1 day and 7 days after the training day. The training session and probe trials were monitored and analyzed using the SMART (Version 2.5.19) real-time video-tracking system.

### Barnes maze

The Barnes maze was executed according to ( Berta *et al.*, 2007). In short, the maze consisted of a circular platform (92 cm diameter; opaque white) with 20 equally spaced holes (5 cm diameter; 7.5 cm between holes) and was elevated 105 cm above the floor. All holes were 2 cm away from the perimeter of the platform and a dark escape box (10 cm × 12.5 cm × 12.5 cm) was located beneath one of the holes.

The mouse was placed in a cylindrical black start chamber (10 cm × 12.5 cm × 12.5 cm) in the middle of the maze. After 10 s the chamber was lifted and the mouse was exposed to bright light (500 W). For the habituation trial on day 1 the animal was gently guided to the escape box and once it entered, the entry hole was covered and the mouse kept for 2 min in the dark escape box. Between each mouse, before the next trial, the platform was cleaned with a 10% Nolvasan solution to avoid remaining olfactory cues. During the acquisition phase, after the start chamber was lifted, the mouse was allowed to explore the maze for 3 min. The trial ended when the mouse entered the escape box or after 3 min had elapsed. Immediately after the mouse entered the escape box, the entry hole was covered and the animal stayed for 1 min in the dark escape box. If the mouse did not reach the target hole/escape box within 3 min, it was gently guided to it. After 1 min in the escape box, the mouse was placed back in its home cage until the next trial. Each mouse received 4 training trials per day with an inter-trial interval of 10–15 min on 4 consecutive days.

The probe trial was conducted on day 5, i.e., 24 h after the last training day, and on day 12. During the probe test the escape box was removed and the mouse was allowed to explore the maze for a fixed time of 90 s. The number of pokes (errors) in each hole and the latency and path length to reach the virtual target hole was measured using the SMART (Version 2.5.19) real-time video-tracking system.

### Elevated plus maze

The elevated plus maze (EPM) was a four arm maze with each arm measuring 30 × 5 cm and the central platform measured 5 × 5 cm (opaque white). One set of arms, opposing one another, were enclosed completely by grey side walls, 15 cm high, while the other set was open with a ledge of 0.5 cm on either side of the arms. The maze was elevated 100 cm from the floor and illuminated evenly with a 500W light. Mice were placed on the central platform, facing towards a closed arm, and allowed to freely explore the maze for 5 min. The SMART (Version 2.5.19) real-time tracking software was used to record the locomotor activity and the time spent on both the closed and the open arms during the test.

### Fear conditioning

Contextual fear conditioning was conducted using the ‘Video Tracking of Fear Conditioning System’ and the ‘Video Freeze Software’ from MED Associates Inc. The experimentally naïve mice were placed in the conditioning chamber and received the first foot shock at the end of a 3 min habituation period. The mice remained for an additional minute in the chamber after the last foot shock was delivered. For the 5 shock conditioning protocol, the mice received an electric foot shock (0.75 mA, 1 s) at the end of the 3 ^rd^, 4 ^th^, 5 ^th^, 6 ^th^, and 7 ^th^ minute. During the milder 3 shock (0.5 mA, 1 s) paradigm, the shock was received at the end of the 3 ^rd^, 4 ^th^ and 5 ^th^ minute. For the 4 day training protocol only one shock (0.75 mA, 1 s) per day was delivered after 2.5 min. In case of the milder 3 shock paradigm and the 4 day training protocol the mice were pre-exposed to the conditioning chamber for 3 min and 10 min respectively. For recall the mice were placed back for 5 min in the same chamber after the indicated periods of time. If the mice were exposed to a different context, the chamber geometry was changed (from square to curved wall), the lights were dimmed, the rod-flooring was covered with white linoleum, and the scent was changed from 0.01% bleach to bubble gum. Using the Video Freeze Program, the freezing time was determined (in %) and was defined as the absence of any movement, except for respiratory motion. The observed freezing response to the foot shock is correlated to the degree of learning, the strength of the aversive stimulus, and the number of presentations ( Curzon *et al.*, 2009).

### Statistical analysis

All data are represented as mean ± the standard error of the mean (SEM). The data were analyzed by unpaired two-tailed t-test, one-way ANOVA or two-way ANOVA followed by the Bonferroni correction as indicated in the results, using GraphPad Prism 5 software. Statistical significance was considered if p ≤ 0.05.

## Results

### Single day spatial learning in the MWM is impaired in GluN2B KI

**Figure 1.**
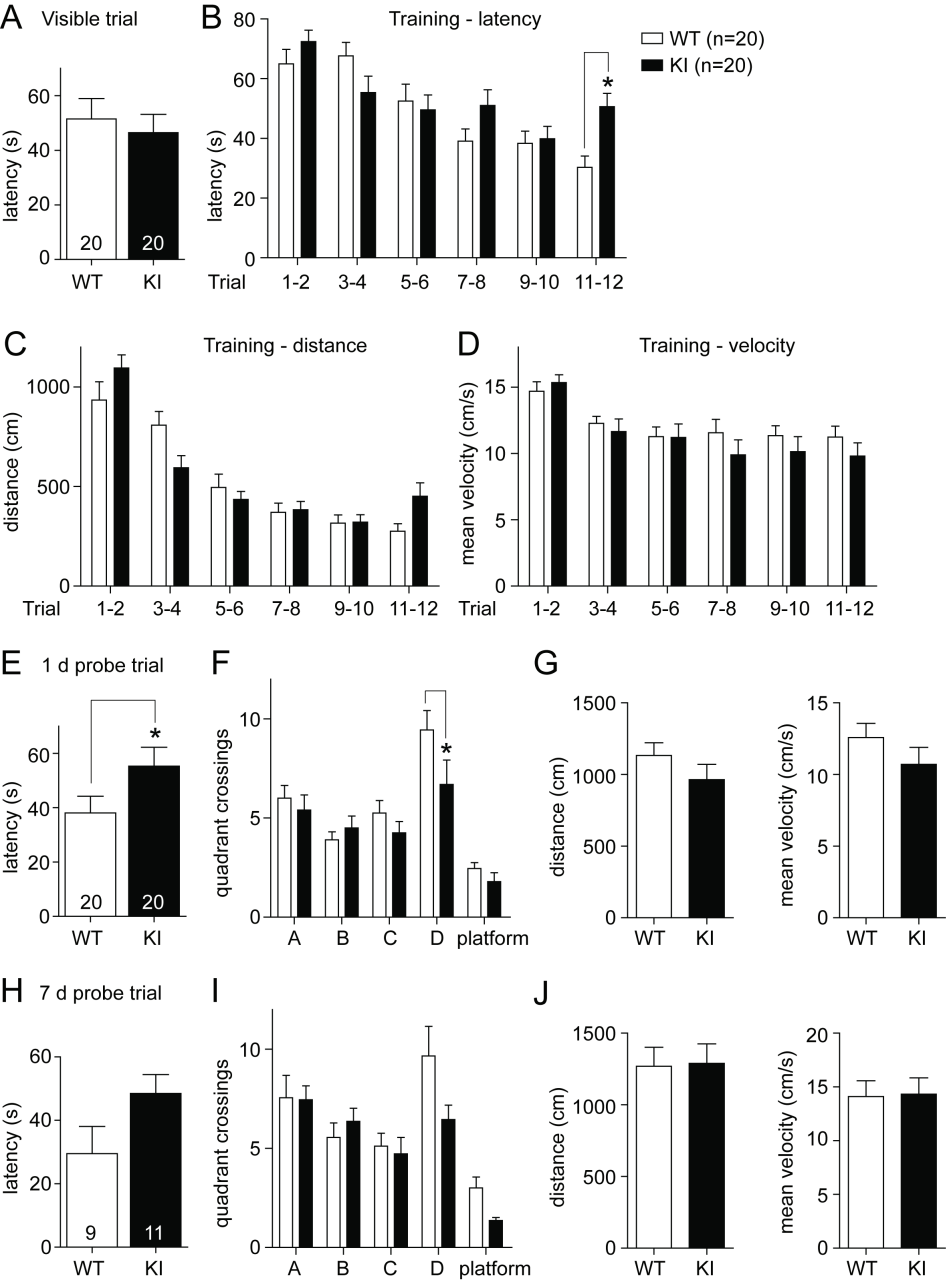
Spatial learning in the Morris water maze (MWM) is impaired in GluN2B KI mice. ( **A**) WT and KI mice display no difference in the average escape latency during the visible platform trial. ( **B**) The GluN2B KI mice show a deficit during acquisition of the hidden platform task. The latencies of the 12 training trials are split up into blocks of two and the average escape latency is significantly increased for KI versus WT mice during the last two training trials. ( **C, D**) The covered distance over the blocks of two training trials is also different between KI and WT mice ( **C**), while the mean velocity is not changed ( **D**). ( **E, F**) During the probe test 1 day after training the KI mice exhibit increased primary average escape latency ( **E**) and show significantly fewer crossings into the target quadrant D ( **F**), the former location of the submerged platform, compared to WT mice. ( **G**) Both genotypes cover the same distance during the probe trial and display the same mean velocity. ( **H, I**) The KI mice show a similar tendency during the probe test 7 days after training, with extended primary average escape latency ( **H**) and diminished crossings into target quadrant D ( **I**). ( **J**) The covered distance and mean velocity is again unchanged. (A: adjacent right quadrant to D; B: quadrant opposite to quadrant D; C: adjacent left quadrant to D; platform: area of the former location of the platform). The same two independent cohorts, each consisting of 10 WT and 10 GluN2B KI mice, were tested in the Barnes maze before the MWM. Data represent Mean ± SEM.

In order to further dissect the memory deficit observed during the initial MWM experiments ( Halt *et al.*, 2012), two independent cohorts of GluN2B KI mice and their WT littermate controls (10 mice of each genotype per cohort) underwent a massed training protocol on a single day in the MWM. Such a compressed training protocol challenges the learning capacity within a short time period. It also has the potential to reveal deficits in early- versus late-phase consolidation as training is not protracted over 6 days as is the case for the classic MWM. The 1 day protocol thus might expose deficits in early consolidation when testing different cohorts 1 and 7 days after the training day without the complication that in the 6 days training paradigm training and consolidation processes overlap. After habituation on the first day (training day), mice underwent one visible platform trial followed by 12 consecutive training trials, in which the platform was no longer visible but kept in a fixed position. The initial trial with a marked visible platform is hippocampus-independent and was conducted to rule out changes in motivation, coordination, or sensory processing in GluN2B KI mice. The latency in reaching the visible platform was not different between the two genotypes ( Figure 1A; WT: 51.48 ± 7.43 s, KI: 46.46 ± 6.70 s). In the following 12 consecutive trials with a submerged platform the KI mice slowly started to show deficits in spatial learning around trial 6, roughly the onset of learning in the WT mice ( Figure 1B). The latencies of the 12 training trials are paired into blocks of two for analysis and the GluN2B KI mice display significantly increased average escape latencies during the last two trials (genotype: F1,228=2.63, p=0.1061; trial: F5,228=13.30, p<0.0001; genotype × trial F5,228=3.08, p=0.0103; Bonferroni post hoc test shows that the latency is significantly increased in the KI mice during the last two trials, M _diff_=20.30 s, 95% Cl [2.58, 38.01], p<0.05; Figure 1B; WT _11–12_: 30.31 ± 3.77 s, KI _11–12_: 50.61 ± 4.49 s).

The covered distance shows like the escape latency a significant *genotype x trial* interaction ( Figure 1C; genotype: F1,228=0.16, p=0.687; trial: F5,228=43.34, p<0.0001; genotype × trial F5,228=3.13, p=0.0095), strengthening the mild learning deficit observed during the last training trials. No difference was observed between the mean velocity of the WT and GluN2B KI mice ( Figure 1D).

During the probe test one day after training, WT mice required significantly less time to reach the original location of the platform as reflected by faster primary escape latency ( Figure 1E; WT: 38.09 ± 6.15 s, KI: 55.32 ± 6.91 s; t(38)=2.15, p<0.05). Analysis of the entries into the quadrants and the area of the former platform location also showed a significant difference between the two genotypes in their preference for the target quadrant ( Figure 1F). The KI mice showed compared to WT no real preference for the target quadrant D and entered it significantly less often ( Figure 1F; WT _D_: 9.45 ± 0.98, KI _D_: 6.70 ± 1.22; genotype: F1,190=3.89, p≤0.05; quadrant/platform: F4,190=18.99, p<0.0001; genotype × quadrant/platform F4,190=1.46, p=0.2147; Bonferroni post hoc test shows that the WT mice search preferentially in the target quadrant compared to the KI mice, M _diff_=-2.750 entries, 95% Cl [-5.347, -0.153], p<0.05). The covered distance and mean velocity was not different between the two genotypes ( Figure 1G), indicating similar activity levels.

During the probe test 7 days after training some of the mice (independent of their genotype) were continuously floating and did not search actively for the escape platform. For the analysis of the 7 day probe trial, these floaters were neglected. The performance in the probe test 7 days after training showed a similar tendency compared to the one day test. The GluN2B KI leaned towards a longer primary escape latency ( Figure 1H, left; WT: 29.50 ± 8.59 s, KI: 48.50 ± 5.98 s; t(18)=1.87, p=0.078) and tended to cross less often over into the target quadrant in search for the platform than WT mice ( Figure 1I; WT _D_: 9.67 ± 1.49, KI _D_: 6.46 ± 0.73; genotype: F1,90=3.12, p=0.0805; quadrant/platform: F4,90=16.69, p<0.0001; genotype × quadrant/platform F4,90=1.85, p=0.1259). Both genotypes again showed no observable difference in the covered distance and mean velocity ( Figure 1J).

Individual analysis (one-way-ANOVA) of the search pattern (quadrant crossings) reveals that the WT mice preferentially search in the target quadrant during the 1 day (F3,76=11.61, p<0.0001) as well as the 7 day probe trial (F3,32=3.91, p=0.0175). The GluN2B KI mice conversely only show a slight non-significant preference for the target quadrant during the 1 day probe trial and no preference at all during the 7 day probe trial.

### Spatial learning and memory is not affected in the Barnes maze

**Figure 2.**
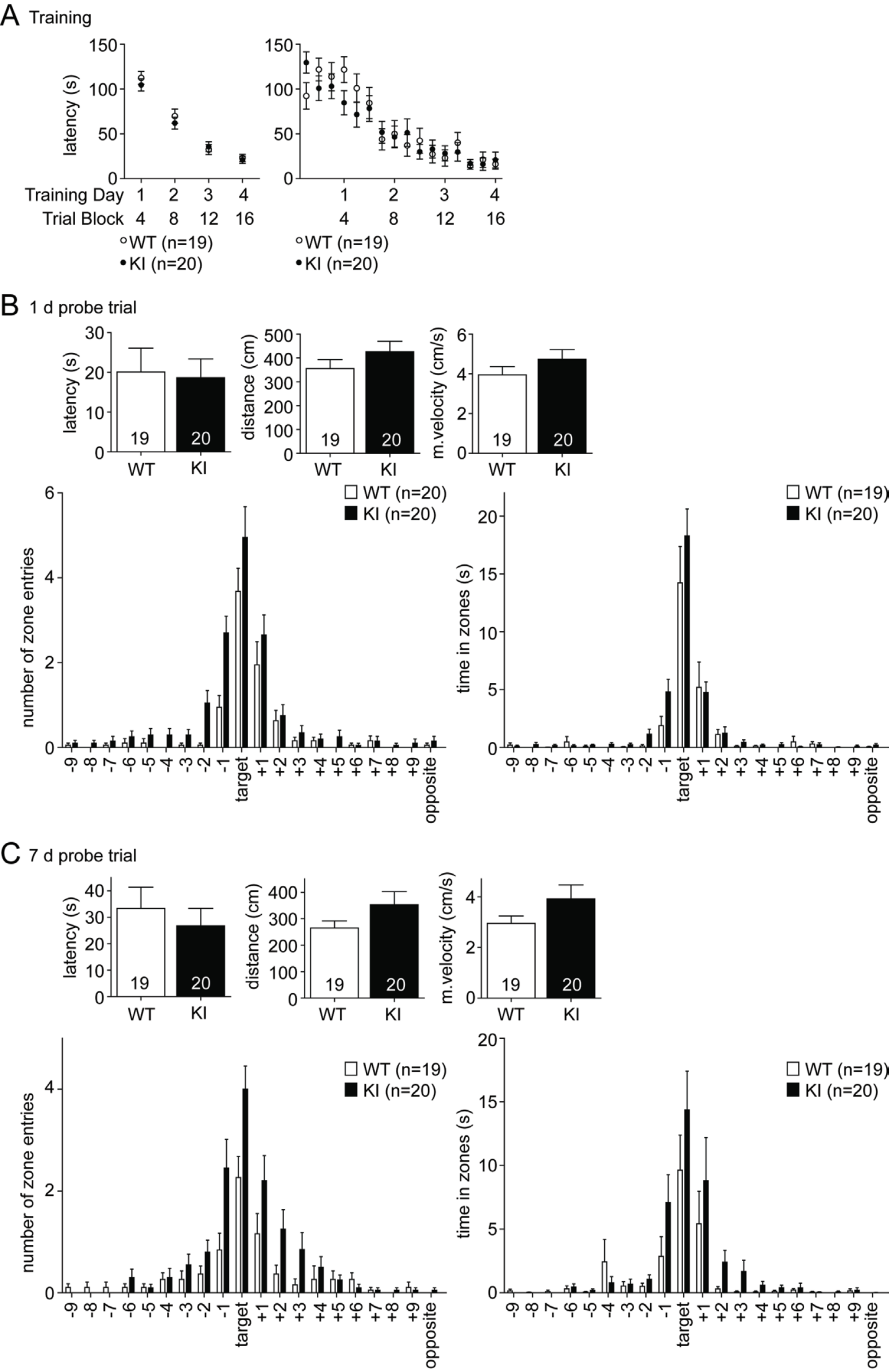
Spatial learning and memory is unaffected in the Barnes maze. ( **A**) The escape latency for both genotypes decreases equally over the 16 training trials and saturates on the fourth day (left panel depicts average latencies over each of the 4 training days whereas right panel shows averages for each individual trial). ( **B**) The primary escape latency during the probe test 1 day after the last training session is not different. KI mice search like the WT mice preferentially in and around the target area for the escape box (bottom panels). The covered distance (middle) and mean velocity (right) is slightly, but not significantly increased in KI mice. ( **C**) The probe test 7 days after the last training session shows, like the 1 day test, no difference in the avg. primary escape latency (left) and the KI mice again exhibited the tendency to increased covered distance (middle) and mean velocity (right). Both genotypes searched in the right location in and around the target area (bottom panels). The same two independent cohorts, each consisting of 10 WT and 10 GluN2B KI mice, were tested in the Barnes maze followed by the MWM. Data are presented as Mean ± SEM.

The Barnes maze is a less aversive spatial learning paradigm, which unlike the MWM allows the mice to move freely and explore the environment under conditions that are less stressful. It is solely based on the motivation of the mice to avoid prolonged exposure to an open area under light and to search for a dark and safe hideout, the escape box. Interestingly, the GluN2B KI showed no detectable deficits not only in learning but also in early memory ( Figure 2). These findings contrast what had been seen before after spaced MWM learning experiments conducted over a period of 6 days ( Halt *et al.*, 2012). Over the 16 training trials, which were executed on 4 consecutive days (4 trials per day) the latency to locate and enter the escape box decreased equally fast for both genotypes and saturated on the fourth day ( Figure 2A; WT: 22.99 ± 4.08 s, KI: 20.95 ± 3.95 s). During the probe test 1 day after the last training session there was no difference in the primary escape latency ( Figure 2B, left; WT: 20.11 ± 6.02 s, GluN2BKI: 18.68 ± 4.72 s), covered distance ( Figure 2B, middle; WT: 355.70 ± 37.25 cm, GluN2BKI: 426.60 ± 43.64 cm) or mean velocity ( Figure 2B, right; WT: 3.95 ± 0.41 cm/s, GluN2BKI: 4.74 ± 0.48 cm/s). Both WT and KI mice searched preferentially in the target area and the surrounding holes (areas +1 and -1), indicating that they clearly remembered the former location of the escape box ( Figure 2B, lower part). The overall number of zone entries and time in zones for GluN2B KI mice showed a slight increase compared to WT mice, which is probably a reflection of the statistically non-significant increased activity of KI as seen in the covered distance and mean velocity ( Figure 2B). The performance in the probe test 7 days after the last training session mirrored the results of the 1 day probe test. There was no significant difference in the primary escape latency ( Figure 2C, left; WT: 33.37 ± 7.98 s, KI: 26.80 ± 6.61 s), covered distance ( Figure 2C, middle; WT: 265.40 ± 26.47 cm, GluN2BKI: 353.00 ± 49.42 cm) and mean velocity ( Figure 2C, right; WT: 2.95 ± 0.29 cm/s, GluN2BKI: 3.92 ± 0.55 cm/s). The slightly increased activity of the GluN2B KI mice is again reflected by an increased number of zone entries and by the time spent in the zones ( Figure 2C, bottom).

### Basal anxiety levels in the GluN2B KI mice are normal

**Figure 3.**
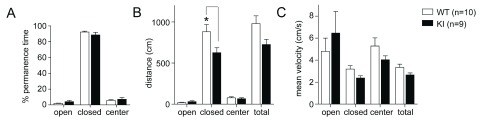
Basal anxiety levels in the GluN2B KI mice are normal. ( **A**) The % of time the mice spent in the open and closed arms as well as the center is not different between WT and KI. ( **B**) Both genotypes cover the same minimal distances in the open arms but WT mice cover greater distances than KI mice in the closed arms. ( **C**) The overall mean velocity is not different between both genotypes, while the WT mice show a slightly increased mean velocity in the closed arm coinciding with the longer covered distance. One cohort of 10 WT and 9 GluN2B KI mice was tested. The same cohort was subsequently used for the milder three shock fear conditioning paradigm ( Figure 5A–E). Data represent Mean ± SEM.

Basal anxiety levels in the GluN2B KI mice were assessed using the EPM. The EPM relies on the rodents’ innate fear of heights and open spaces and their preference for dark and enclosed rooms. Basal anxiety levels in the GluN2B KI mice were not different compared to their WT litter-matched controls. During exposure to the EPM the GluN2B KI mice spent most of the time in the closed arms, like the WT controls, and rarely stayed in the open arms or center ( Figure 3A; in % of total time, WT: 92.30 ± 1.16%, KI: 88.68 ± 3.27%). Both genotypes covered comparable minimal distances in the open arms ( Figure 3B; WT: 20.63 ± 3.96 cm, GluN2B KI: 31.62 ± 10.73 cm), while the WT mice had the tendency to be more active in the closed arms. This tendency is displayed in an increase in covered distance ( Figure 3B; WT: 881.59 ± 86.40 cm, KI: 626.92 ± 59.24 cm; genotype: F1,68=10.17, p=0.0022; location: F3,68=120.21, p<0.0001; genotype × location F3,68=3.40, p=0.0225; Bonferroni post hoc test shows that the distance is significantly reduced in the KI in the closed arms, M _diff_=-254.7 cm, 95% Cl [-459.5, -49.49], p<0.01) and a trend towards a higher mean velocity in the closed arms ( Figure 3C; WT: 3.19 ± 0.31 cm/s, GluN2B KI: 2.37 ± 0.21 cm/s), also apparent in the overall mean velocity ( Figure 3C; WT: 3.34 ± 0.29 cm/s, GluN2B KI: 2.66 ± 0.18 cm/s). The observed normal basal anxiety levels concur with earlier findings in the open field analysis and the same innate fear reaction to trimethyl-thiazoline (TMT) ( Halt *et al.*, 2012).

### Learning and memory in contextual fear conditioning is not affected in GluN2B KI mice

**Figure 4.**
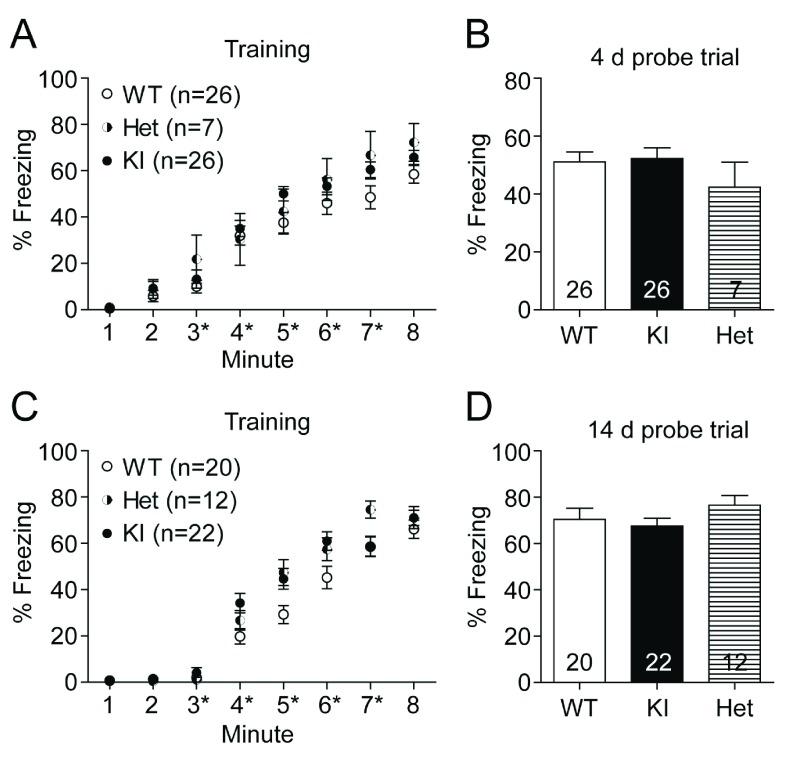
Contextual fear conditioning and memory is unaffected in GluN2B KI mice. Fear conditioning was evaluated 4 days after training of two different cohorts of naïve litter-matched WT, heterozygous, and GluN2B KI mice (12–14 WT and KI mice, and 3–4 heterozygous mice per cohort) and 14 days after training with yet another two different cohorts of naïve litter-matched WT, heterozygous, and GluN2B KI mice (10 WT mice, 10–12 KI mice, and 5–7 heterozygous mice per cohort). ( **A, C**) After the 3 min habituation phase, 5 shocks (0.75 mA, 1 s) were delivered at the end of 3 ^rd^, 4 ^th^, 5 ^th^, 6 ^th^ and 7 ^th^ minute (asterisks). The increase in the fraction of time spent freezing during conditioning is not different between the GluN2B KI, heterozygous, and WT mice. ( **B, D**) All genotypes also display a comparable fraction of time freezing during the 4 day and the 14 day probe test. Data represent percentage freezing (Mean ± SEM).

With basal anxiety levels not being affected in the GluN2B KI mice ( Halt *et al.*, 2012) ( Figure 3), we investigated the role of the CaMKII/GluN2B interaction in contextual fear conditioning. The experimentally naïve mice were placed in the conditioning chamber and after a 3 min habituation period trained with five consecutive foot shocks (0.75 mA, 1 s duration; aversive unconditioned stimulus). The GluN2B KI mice learned to the same extent and at the same speed as their WT litter-matched controls ( Figure 4A, C). A total of four independent cohorts were trained. To be able to distinguish possible differences between memory recall and consolidation, two cohorts were tested after 4 days ( Figure 4B) and the other two cohorts after 14 days ( Figure 4D). For the probe tests (after 4 or 14 days) the mice were placed back for 5 min into the conditioning chamber and the freezing response to the context was measured. There was no difference in the average time WT and KI mice spent freezing during either the 4 (WT: 51.11 ± 3.37%, KI: 52.23 ± 3.77%) or 14 days (WT: 70.48 ± 4.75%, KI: 67.51 ± 3.33%) probe test.

### Contextual fear conditioning performance is independent of the stimulus strength

CaMKII T286A mutant mice have impaired contextual short-term memory (STM) and long-term memory (LTM) formation after a single or three tone-shock pairings, while contextual STM and LTM formation after five pairings are unaffected ( Irvine *et al.*, 2011; Irvine *et al.*, 2005). To exclude the possibility that the GluN2B KI mice were over trained with five consecutive foot shocks (0.75 mA, 1 s duration) and potential cognitive deficits masked, we also tested two milder contextual fear conditioning paradigms.

**Figure 5.**
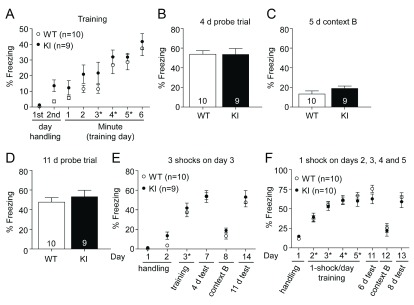
Contextual fear conditioning with milder conditioning is unaffected in GluN2B KI mice. One cohort of litter-matched GluN2B KI and WT mice (9–10 mice per genotype, previously tested in the EPM) was fear conditioned with three shocks on a single day and a different cohort of naïve litter-matched GluN2B KI and WT mice (10 mice per genotype) was fear conditioned with four shocks over a period of 4 days. ( **A**) KI and WT mice acquired to the same extent fear conditioning as indicated by the time spent freezing after three consecutive mild foot shocks (0.5 mA, 1 s) at the end of the 3 ^rd^, 4 ^th^ and 5 ^th^ minute (asterisks). ( **B, D**) The time spent freezing during recall of context after 4 d and 11 d is not different between genotypes. ( **C**) Exposure to a different context 5 days after training shows pre-conditioning freezing levels for both genotypes. ( **E**) Summary plot of the experimental data from A-D. ( **F**) Summary plot of the experimental data of the 4 day training (1 shock, 0.75 mA, 1 s per day) experiment. WT and KI mice show comparable learning curves during the 4 d conditioning and similar contextual memory when tested 6 and 8 days later. Exposure to a different context in the same room and chamber, 7 d after the last training day, revealed again pre-conditioning freezing levels. Data represent percentage freezing (Mean ± SEM).

During the first paradigm, after two days of 3 min pre exposure to the conditioning chamber, 3 shocks (0.5 mA, 1 s) were delivered on day 3 at the end of the 3 ^rd^, 4 ^th^ and 5 ^th^ minute. Conditioning and memory tested after 4 days (WT: 53.82 ± 3.55%, KI: 53.55 ± 6.10%) were unaffected ( Figure 5A, B). When the mice were exposed to a different context in the same chamber and room 1 day later, the time spent freezing was reduced, similar to pre-conditioning levels, and did not differ between the two genotypes ( Figure 5C; WT: 13.33 ± 3.20%, KI: 18.90 ± 2.55%). Long term memory was tested with the same cohort 11 days after training ( Figure 5D) and like the 4 day test showed no difference (WT: 47.63 ± 4.60%, KI: 53.08 ± 6.60%). Figure 5E depicts an overview of the experiment.

In addition, another cohort of GluN2B KI and WT mice were trained over multiple days. The mice were pre exposed to the context on day 1 for 10 min and trained on the following 4 days with one foot shock (each 0.75 mA, 1 s) per day ( Figure 5F). Again no learning or memory deficit, which was tested 6 (WT: 75.52 ± 4.64%, GluN2B KI: 62.69 ± 6.21) and 8 days (WT: 64.75 ± 5.68%, KI: 59.06 ± 7.56%) after training, was observed. When the mice were exposed to a different context in the same chamber and room on day 12, 7 days after the last training, both genotypes exhibited reduced freezing levels (WT: 25.56 ± 5.85%, KI: 22.09 ± 6.93%) similar to those observed before conditioning.

Data sets for behavioral tasks in wild type and GlunN2BKI mice The raw data tables show the performances of the individual wild type and GlunN2B KI mice in the various behavioral tasks, which are represented in the text and figures as mean ± the standard error of the mean (SEM). Data sets for ‘MWM data’, ‘Barnes maze data’, ‘Elevated plus maze data set’ and ‘Contextual fear conditioning data set’ are shown. Specific information can be found in text file.

## Discussion

CaMKII and the NMDAR are crucial not only for hippocampal LTP but also for hippocampus dependent spatial learning and memory formation. In a number of studies CaMKIIα KO and mutant mice displayed learning deficits, notably mainly in aversively motivated tasks ( Elgersma *et al.*, 2002; Giese *et al.*, 1998; Irvine *et al.*, 2005; Silva *et al.*, 1992), while appetitive learning was unaffected ( Carvalho *et al.*, 2001). Moreover, the regulation of expression or activity level of CaMKIIα seems to have an emotional component and especially impact emotional and anxiety-like behavior and learning. CaMKIIα heterozygous KO mice show a decreased freezing in fear conditioning induced by an electric foot shock and are more active in the open field suggesting decreased anxiety-related behaviors ( Chen *et al.*, 1994). Consistently, transgenic mice overexpressing CaMKIIα exhibit an increase in anxiety-like behaviors in open field, light-dark transition, and elevated zero maze ( Hasegawa *et al.*, 2009). CaMKIIα T286A mutant mice, which are deficient in autonomous and thereby overall CaMKII activity, spend more time in and entered more often the open arm in the EPM and generally react with hyperactivity to novel stimuli that could be perceived as potentially threatening ( Easton *et al.*, 2011). However, the behavior in the non-threatening neutral environment of the home cage is normal ( Easton *et al.*, 2011), in accordance with an impaired learning in MWM and fear conditioning ( Giese *et al.*, 1998; Irvine *et al.*, 2005) but a normal appetitive instrumental conditioning ( Carvalho *et al.*, 2001). Results in our study concur with these findings and argue for the specific importance of CaMKII binding to GluN2B in aversive and more demanding spatial learning task of the MWM, with the less threatening and stressful Barnes maze task being unaffected in the KI mice. Contextual fear conditioning and memory was surprisingly not affected in the GluN2B KI mice. We assume that this discrepancy is due to the higher demands of the MWM task and the differential regulation of spatial and contextual LTM formation ( Mizuno & Giese, 2005).

Learning during massed training sessions, i.e., when training trials are given in short order on a single day, generally results in lower levels of learning than spaced training over a period of several days. For instance, in the MWM rats showed faster and better acquisition during spaced (16 trials over 4 days) than massed training (16 trials in 1 day) as well as better memory formation ( Commins *et al.*, 2003). During contextual fear conditioning 1 h spaced training also results in better memory formation than massed training, while cued fear conditioning is unaffected ( Scharf *et al.*, 2002). Likely because of the more demanding task of massed training, which requires information being stored during a shorter time period than in spaced training, the GluN2B KI mice revealed a mild learning deficit compared to WT mice in the 1 day paradigm ( Figure 1) when they did not in the 6 day MWM training protocol ( Halt *et al.*, 2012). Figure 1D shows that there is no difference in the swim speed between WT and KI mice, which together with the increased covered distance of the KI mice during the last two trials ( Figure 1C) argues against the differences in agility and fatigue in the GluN2B KI. The increased covered distance shows that the KI mice have to swim longer and cover a greater distance in order to find the platform during the later trials, further supporting the extended primary escape latency ( Figure 1B) and therefore the KI learning deficit. The learning deficit in the 1 day paradigm suggests that the learning capacity over a more limited time period is reduced in GluN2B KI mice. In addition, the elevated stress level during the massed training in the 1 day protocol could affect learning in the GluN2B KI mice more than in WT contributing to the differential outcome.

The GluN2B KI phenotype is similar to alpha and delta CREB KO mice, which are impaired in massed but not in spaced MWM learning and contextual fear conditioning ( Kogan *et al.*, 1997). The improvement in memory after spaced learning is also mimicked in acute hippocampal slices where successive spaced theta burst stimulation resulted in enhanced previously saturated LTP ( Kramar *et al.*, 2012). In the study by Kramar *et al.* the induction of LTP in a subset of synapses during the first theta burst train primed the initially unresponsive neighbors (probably in a translation-dependent manner, since the two stimulations have to be spaced 1 h apart; see also ( Scharf *et al.*, 2002)), resulting in potentiation after stimulation with the second theta burst train. This mechanism will probably be especially important in cases of impaired LTP induction or expression like in our GluN2B KI mice where various forms of LTP were reduced by ~50% ( Halt *et al.*, 2012).

The Barnes maze is a demanding spatial task using visual cues in the room for navigation, which is in that regard similar to the MWM. One major difference between these two spatial learning paradigms is probably the amount of stress to which animals are exposed. The unaffected performance of the GluN2B KI mice in the less stressful Barnes Maze is in line with the known importance of CaMKII selectively in aversive rather than appetitive motivated spatial and contextual learning paradigms ( Elgersma *et al.*, 2002; Giese *et al.*, 1998; Irvine *et al.*, 2005; Silva *et al.*, 1992). However, further potential differences include sensory processes and response requirements, which cannot be excluded as a reason for the deficit in massed MWM learning compared to no deficit in the Barnes maze ( Figure 2).

Fear conditioning, on the other hand, creates a high level of anxiety. Compared to spatial learning paradigms like the MWM, the contextual conditioning only requires memorization of a far less complex environment, which only has to be recognized and does not require navigation and localization of a certain target. Additionally, at the beginning of the conditioning the mice have time to explore and form a spatial map without being stressed or more anxious than during the normal exposure to a novel environment, before the first shock is delivered at the end of the 3 ^rd^ minute. During the milder conditioning paradigm ( Figure 5) the mice were pre-exposed to the shocking chamber on the previous day, which allowed the formation of an even better spatial representation ( Fanselow, 2000; Frankland *et al.*, 2004; Hu *et al.*, 2007). This lack of a demanding spatial learning requirement and possible formation of contextual memory during still low stress levels could explain the normal conditioning and contextual memory ( Figure 4 and Figure 5) compared to the moderate learning deficit observed during massed MWM training ( Figure 1). In addition, fear conditioning in general constitutes a very strong learning paradigm, which might override and mask any mild learning deficits because of its vigor even in the mildest form tested here. In support of this idea the five tone-shock pairings allow full fear conditioning in CaMKII T286A mutant mice when one and three pairings do not ( Irvine *et al.*, 2011; Irvine *et al.*, 2005). Accordingly, the strength of conditioning can mask learning deficits, and for GluN2B KI mice that might even be the case for single shock experiments. Spatial and contextual LTM formation is differently regulated. There are not only divergent regional requirements within the hippocampus, but also the control of gene transcription varies between spatial and contextual LTM formation ( Mizuno & Giese, 2005). Accordingly, MWM and fear conditioning could depend on molecular mechanisms that vary in detail even though both learning paradigms require CaMKII.

Contrasting CaMKIIα heterozygous KO, T286A KI mice, or transgenic mice overexpressing CaMKIIα, the GluN2B KI mice display no change in basal anxiety levels or anxiety-like behaviors. They show avoidance of the open arms in the EPM ( Figure 3), behavior in the open field ( Halt *et al.*, 2012), and reaction to TMT (the anxiogenic compound in fox urine) ( Halt *et al.*, 2012), a measure of innate fear, that are comparable to WT mice. Thus it appears that CaMKIIα affects fear behavior independent of its binding to GluN2B.

Our study suggests a specific requirement for the activity-dependent interaction of CaMKII with the NMDAR during the acquisition of more elaborate spatial learning tasks under modestly aversive, stressful conditions. The results argue not only for a role of CaMKII binding to GluN2B during consolidation ( Halt *et al.*, 2012), but also for learning, which becomes obvious under the here applied more demanding MWM conditions. The less stressful Barnes maze neither results in a consolidation deficit nor is the learning itself affected. In addition, the less demanding but more potent contextual fear conditioning and memory was surprisingly normal. Whether reduced recall of the MWM task 1 and 7 days after single day training reflects reduced learning, reduced memory consolidation, or both cannot be answered at this point.

## Data availability

F1000Research: Dataset 1. Data sets for behavioral tasks in wild type and GlunN2BKI mice, 10.5256/f1000research.4660.d34165 ( Stein *et al.*, 2014).
