# Supplementary material for: CaMKII binding to GluN2B is important for massed spatial learning in the Morris water maze
Source: F1000Res. 2014 Aug 12;3:193. [Version 1] doi: 10.12688/f1000research.4660.1 (PMC4149248; doi:10.12688/f1000research.4660.1)
Supplement: Peer review history

# Peer review: Referee response for version 1

**DOI:** 10.5256/f1000research.4981.r5919

**Published:** 2014-08-26

**Authors:** Karl-Ulrich Bayer

**Affiliations:** 1 Department of Pharmacology, University of Colorado Denver, Aurora, USA

**Summary:** This study substantially extends the previous behavioral analysis of a mouse line with mutant GluN2B that is incapable of CaMKII binding. Individually, both GluN2B and CaMKII are critically involved in regulating synaptic plasticity and learning and memory. Previous studies from the same lab showed that the mutant GluN2B KI mice have impaired hippocampal CA1 LTP (reduced to ~50%) and impaired recall during the consolidation phase of a Morris water maze (MWM) task ^1^. Here, learning impairments were found in a modified MWM task that involves massed learning during day 1. All other learning paradigms tested here or previously were normal. Basal behavior was also extensively evaluated, and was found to be normal in all tasks tested here or previously.

**Evaluation:** The manuscript is very well written. The experiments and the analyses are solid. The interpretation and discussion of the results are both careful and insightful. Overall, it can be very reasonably concluded that learning requires the CaMKII/GluN2B interaction only when the learning task is rather difficult and/or involves some stress (such as in a water maze), consistent also with impaired but not abolished LTP in these mice.

**Comments:** The conclusions from this study may also inform potential future behavioral studies on these mice. For instance, while the GluN2B KI mice were normal in multiple variations of contextual fear conditioning, the contextual part of the task was made rather easy in all paradigms tested. However, even with shorter exposure to the context, fear conditioning may still be too easy of a learning task to reveal any differences in the GluN2B mice. Thus, while such future studies may be interesting, either outcome would still be consistent with the conclusions reached here.

The behavioral analyses of the GluN2B KI mice were done specifically in order to evaluate the impact of the LTP impairments in these mice. When comparing the GluN2B KI mice with CaMKII T286A or CaMKII knock-out mice in other behavioral tasks in future studies, it may have to be taken into account that the GluN2B KI mice are impaired only in LTP, while the CaMKII mutant mice are additionally impaired in LTD.

**One point in which my opinion differs** is within the introduction, specifically regarding the statement that *“auto-phosphorylation on T286 is required for effective binding of CaMKII to GluN2B”*. The cited references could indeed be interpreted support this statement ^2^ ^3^. However, newer data indicate that the increased binding observed after ATP addition was largely caused by direct nucleotide binding to the kinase, rather than by auto-phosphorylation ^4^ ^5^ ^6^.

Notably, this point does not affect the validity of any conclusions reached in the manuscript. However, it may significantly mislead the reader about the larger context of CaMKII in synaptic plasticity. Recently, we have shown that CaMKII T286 phosphorylation is required not only for LTP, but also for LTD ^7^. Prior to this, other recent evidence had already suggested a more prolonged T286 phosphorylation after LTD than after LTP ^8^ ^9^ ^10^ (for review see Coultrap & Bayer, 2012 ^11^). If T286 phosphorylation would induce the most effective CaMKII binding to GluN2B (as stated), binding would be expected to be more extensive after LTD compared to LTP. However, this does not appear to be the case ^9^, and the CaMKII/GluN2B interaction contributes exclusively to the CaMKII functions in LTP but not in LTD ^1^.

I have read this submission. I believe that I have an appropriate level of expertise to confirm that it is of an acceptable scientific standard.

## References

[1] HaltARDallpiazzaRFZhouYSteinISQianHJunttiSWojcikSBroseNSilvaAJHellJW: CaMKII binding to GluN2B is critical during memory consolidation.*EMBO J*.2012;31(5) : 1931203-121610.1038/emboj.2011.48222234183PMC3297991

[2] BayerKUDe KoninckPLeonardASHellJWSchulmanH: Interaction with the NMDA receptor locks CaMKII in an active conformation.*Nature*.2001;411(6839) : 193801-80510.1038/3508108011459059

[3] StrackSColbranRJ: Autophosphorylation-dependent targeting of calcium/ calmodulin-dependent protein kinase II by the NR2B subunit of the N-methyl- D-aspartate receptor.*J Biol Chem*.1998;273(33) : 19320689-2069210.1074/jbc.273.33.206899694809

[4] BarcombKCoultrapSJBayerKU: Enzymatic Activity of CaMKII Is Not Required for Its Interaction with the Glutamate Receptor Subunit GluN2B.*Mol Pharmacol*.2013;84: 193834-84310.1124/mol.113.08904524056996PMC3834147

[5] O'LearyHLiuWHRorabaughJMCoultrapSJBayerKU: Nucleotides and phosphorylation bi-directionally modulate Ca2+/calmodulin-dependent protein kinase II (CaMKII) binding to the N-methyl-D-aspartate (NMDA) receptor subunit GluN2B. *J Biol Chem*.2011;286(36) : 19331272-3128110.1074/jbc.M111.23366821768120PMC3173099

[6] RobisonAJBartlettRKBassMAColbranRJ: Differential modulation of Ca2+/calmodulin-dependent protein kinase II activity by regulated interactions with N-methyl-D-aspartate receptor NR2B subunits and alpha-actinin.*J Biol Chem*.2005;280(47) : 19339316-3932310.1074/jbc.M50818920016172120

[7] CoultrapSJFreundRKO'LearyHSandersonJLRocheKWDell'AcquaMLBayerKU: Autonomous CaMKII mediates both LTP and LTD using a mechanism for differential substrate site selection.*Cell Reports*.2014;6(3) : 193431-43710.1016/j.celrep.2014.01.00524485660PMC3930569

[8] LeeSJEscobedo-LozoyaYSzatmariEMYasudaR: Activation of CaMKII in single dendritic spines during long-term potentiation.*Nature*.2009;458(7236) : 193299-30410.1038/nature0784219295602PMC2719773

[9] MarsdenKCShemeshABayerKUCarrollRC: Selective translocation of Ca2+/calmodulin protein kinase IIalpha (CaMKIIalpha) to inhibitory synapses.*Proc Natl Acad Sci U S A*.2010;107(47) : 19320559-2056410.1073/pnas.101034610721059908PMC2996683

[10] MockettBGGuevremontDWutteMHulmeSRWilliamsJMAbrahamWC: Calcium/calmodulin-dependent protein kinase II mediates group I metabotropic glutamate receptor-dependent protein synthesis and long-term depression in rat hippocampus.*J Neurosci*.2011;31(20) : 1937380-739110.1523/JNEUROSCI.6656-10.201121593322PMC6622612

[11] CoultrapSJBayerKU: CaMKII regulation in information processing and storage.*Trends Neurosci*.2012;35(10) : 193607-61810.1016/j.tins.2012.05.00322717267PMC3461103

# Peer review: Referee response for version 1

**DOI:** 10.5256/f1000research.4981.r5845

**Published:** 2014-08-22

**Authors:** Mark L. Dell’Acqua

**Affiliations:** 1 Department of Pharmacology, University of Colorado Denver, Denver, USA

This is an interesting article that provides additional, detailed behavioral analysis of hippocampal-dependent spatial and contextual learning and memory performance of knock-in mice that are deficient in CaMKII binding to the GluN2B subunit of the NMDA-subtype ionotropic glutamate receptor. The current findings extend those of the authors in Halt *et al*. (2012) where spaced training over 6 days in the Morris Water Maze (MWM) revealed no deficits in spatial learning but did find significant deficits in consolidation of long-term memory probed several days after training in theses CaMKII binding-deficient GluN2B knock-in mice.

Interestingly, here, using a more intense and demanding one day, massed training version of the MWM, the authors now detect modest deficits in these GluN2B knock-in mice, not only in memory probed one day after training, but also in spatial learning measured on the day of training. However, other single day training spatial/contextual learning and memory tests, that are both more aversive but less spatially challenging (contextual fear conditioning) and less aversive but more spatially challenging (Barnes maze), did not reveal any difference in performance between WT and GluN2B knock-in mice. Thus, an important conclusion of this study is that increased postsynaptic targeting of CaMKII and the increased level of LTP (~50%; see Halt et al.) that are achieved through CaMKII binding to GluN2B are only required for spatial learning when the task is both fairly demanding and at least moderately aversive. These findings should give the field a heightened appreciation that highly-compartmentalized regulation of signaling pathways by postsynaptic protein-protein scaffolding interactions, such as achieved through CaMKII binding to GluN2B during LTP-induction, may not be absolutely essential for spatial learning and memory in general but may have evolved to allow animals to specifically sustain optimal spatial learning and memory capabilities even under demanding and stressful circumstances.

Overall, the manuscript is very well written and the data are rigorously analyzed and presented clearly.

I have read this submission. I believe that I have an appropriate level of expertise to confirm that it is of an acceptable scientific standard.
